# Remembering John Q Trojanowski, in his own words: A life dedicated to discovering building blocks and using them to build bridges of knowledge, collaboration, and discovery

**DOI:** 10.1038/s41531-022-00310-1

**Published:** 2022-04-12

**Authors:** Hilal A. Lashuel

**Affiliations:** Laboratory of Molecular and Chemical Biology of Neurodegeneration, Brain Mind Institute, École polytechnique fédérale de Lausanne (EPFL), Lausanne, Switzerland

**Keywords:** Events, Scientific community

During the past weeks, scientists, academics, students, organizations, and patient groups from all over the world have been mourning the loss of Prof. John Q. Trojanowski. In their own words, he is a legend, an icon, a visionary leader, a titan, a giant, a towering figure, and a larger-than-life figure in the field of neurodegenerative diseases (NDDs). He is also remembered as brilliant, a truly classy man, a warm-hearted and caring mentor, a wonderful and inspirational scientist, and a kind friend. While reading all these comments, tributes, and memorable moments from former students, trainees, and colleagues and reflecting on John’s work accomplishments, I struggled with the one word: loss. How could you mourn the loss of someone who is still giving, teaching, inspiring, and shaping the lives and work of so many people in so many ways?

John’s ongoing charity is the various research centers, institutes, training programs, consortia, and initiatives that he led and developed until the last days of his life. All of these will continue to serve scientists and patients and pave the way for novel therapies to treat NDDs. The knowledge he has produced and shared in the form of publications, lectures, and innovative tools and disease models are today integral components of the cornerstones of the knowledge foundation that we stand on and the launchpads for future transformative discoveries and innovations. Over the past five decades, John and Virginia cultivated and nurtured an amazing and productive scientific family. They have adopted, trained, mentored, inspired, and touched the lives of hundreds of young scientists who will champion his principles and continue his mission of building the road to cures for NDDs one brick at a time.

I struggled to find the best way to honor John and describe his work and achievements in ways that will do him justice. After reading some of his interviews and hearing some of the beautiful analogies he used to communicate complex concepts in NDDs and his views on different topics and controversies, I realized no one is more qualified to tell the world about John than John, with only one exception his life and work partner, Dr. Virginia Lee.

The text below is my humble attempt to use John’s and occasionally Virginia’s words to provide a glimpse of his vibrant personality, research philosophy, and some of the guiding principles and hypotheses that defined his work and contributions to the field of NDDs.

I tried to capture key moments and quotes that provide a window into John’s personality, his personal and scientific journey, his position on many of the controversial topics that the field of NDDs has been debating and wrestling with during the past decades, and his vision for the future of the field. Many of the quotes used here are taken primarily from previously published interviews and profiles of John and Virginia, including an interview by Keith A. Crutcher from Alzforum (1998)^1^, a Nature article profiling both scientists and their work (2006)^2^, an extensive story and interview by Clarivate (2011)^3^, a story published in Penn Today (2016)^4^, and a discussion that took place during the CurePSP Panel discussion on “The Theory of Everything”^5^.

## John’s other half and partner, Virginia M. Lee

**Fig. 1:**
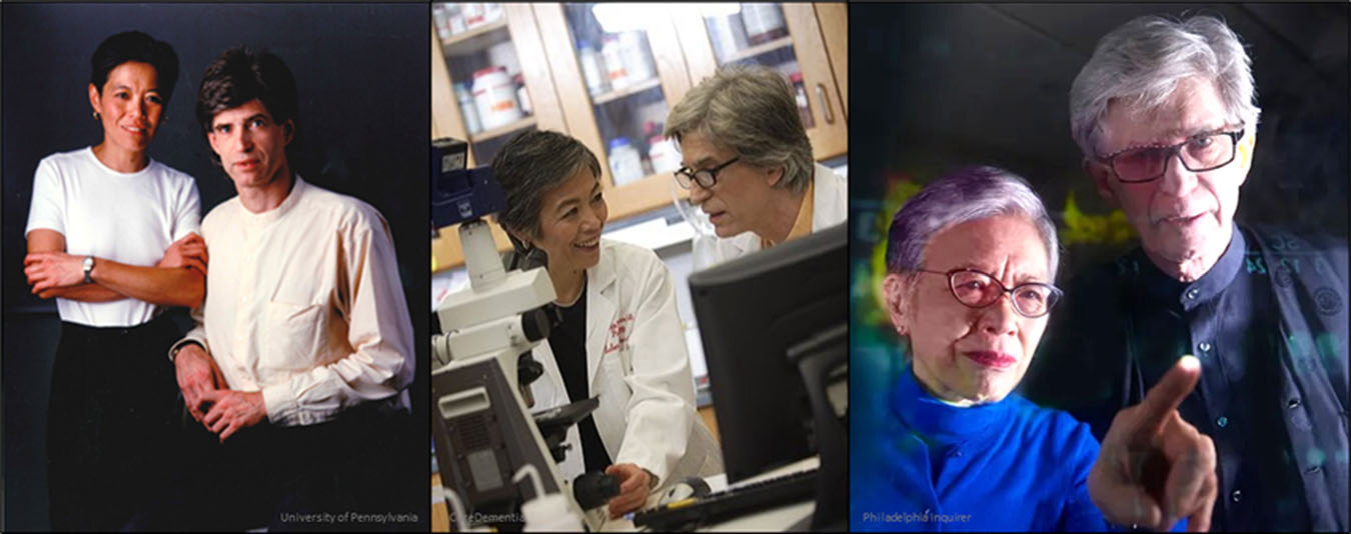
John Trojanowski and Virginia Lee through the years. “John Trojanowski was my life partner and scientific partner and I had the privilege of spending the last 45 years working with him on neurodegenerative diseases. I would not have succeeded in science if it was not for his enthusiasm, his encouragement, his passion in science, and his insistence that together we can get the job done!” —Virginia Lee.

You cannot speak about John without speaking about his life partner, Virginia Lee, and you cannot speak about Virginia without speaking about John, because neither one of them ever did (Fig. 1). John and Virginia have been married since 1979 and collaborating since 1982. They first met in a Boston bar in 1976 while she was a postdoctoral fellow at Boston Children’s Hospital, and he was starting his residency in pathology at Harvard University. Here is how Virginia recalls their first encounter:

“There was this fellow sitting opposite me, and there was something about him that intrigued me,” she said. “My heart skipped a beat. I thought, ‘I just want to get to know this fellow… The rest is history.’”.

John recalled: “I was plaque- and tangle-free”.

The academic system back then and still now discourages scientist couples from working together for the fear that it would be difficult to know the relative contribution of each person and uncouple their achievements during evaluations for tenure and promotions. Despite this, John and Virginia knew that cracking the Alzheimer’s disease (AD) code required team efforts and collaborations between scientists with complementary expertise and skill sets.

“I am a neuropathologist and she is a biochemist. We play together and have a great time.”^4^—John Trojanowski

**They were the perfect team armed with all the skills and expertise to get the job done**, a neuropathologist and neuroanatomist (John) and a biochemist and cell biologist (Virginia). In addition to their complementary expertise, they shared a passion and the determination to achieve an understanding of NDDs that would enable the development of new therapies and disease-modifying strategies. They realized from the beginning that “**the issue is in the tissue”** and that understanding NDDs starts by understanding what happens in the brain, especially from those affected by the disease.

“When we study a protein that underlies a neurodegenerative disease, we study the normal function and the abnormal, deleterious properties that the pathological form of the protein acquires in the disease state. And John and I recognized, back in the beginning of our work together, that to understand these diseases, you really have to start with the human brain. In those days when we started out, in the 1980’s, the science people were doing at the time was not very rigorous for a number of reasons, including a very small ‘tool kit’ for discovery research. There was a lot of room for Ph.D.‘s like me and John to contribute.”^3^—Virginia Lee

## The two-body solution: when one plus one is greater than two

“In his tribute piece to Dr. John Q. Trojanowski, Dr. Hilal Lashuel highlights, through John and Virginia’s own words, the importance of scientific partnerships. Nearly all of us know or can imagine how challenging it can become when the scientific partner is also a life or domestic partner. While academia often refers to this scenario as the ‘’two-body problem”, I am convinced through personal and professional experience that in some cases it can also be a “two-body solution” and Lee and Trojanowski’s life-long scientific partnership is a perfect example of this. Perhaps serendipitously the word cloud generated for John has “Virginia” solidly planted on “John” (Fig. 3) and no one has any doubt that his legacy will be continued by his remarkable partner for years to come. Despite a great deal of progress in the past 20 years, every woman in STEM knows the struggle we still face for equal opportunity and to have our voices respected and recognized. Yet John and Virginia recognized and respected each other’s place in their partnership and always celebrated their scientific achievements as equals. They did so 40 years ago and until his passing, which is another testament to them being way ahead of their time and leading by example.

In memory of the late John Q. Trojanowski, let us honor his achievements and legacy through recognizing and promoting team science and supporting and inspiring the next generation of team scientists so that we can achieve our mutual goal of eliminating age-related neurodegenerative diseases in our lifetime.”—**Malu Tansey**

## Putting a molecular face to the pathology of NDDs

Together, they embarked on a journey to unmask the molecular facets of the pathology of NDDs and decipher their building blocks.

*“…*we like to think one of our major contributions to the field of neurodegenerative diseases is putting a molecular face on these pathology structures that have been known for many, many years, but without information on their building blocks.*“*^3^—John Trojanowski

## The issue is in the tissue

They believed that identifying the building blocks of the proteinaceous inclusions and deposits found in the brain of individuals affected by neurodegenerative diseases was an essential first step to understanding the molecular mechanisms that drive these diseases. The second step was always creating disease models to decipher the key molecular changes and mechanisms underpinning the pathogenesis of NDDs. These models, combined with a better understanding of the biochemical and cellular properties of these building blocks, have paved the way for developing and testing various strategies to treat NDDs, many of which are in clinical trials today.

“Having a molecular specificity is so critical to every next step of solving these diseases because once you have a name, i.e., the protein identity of the building blocks of neurodegenerative disease pathologies, you know what it will be doing, and then it is possible to create a roadmap for targeted drug discovery for these disorders.”^3^—John Trojanowski

## Finding a treasure box in the swamp: a lesson for all young scientists

In many of his interviews, John describes how colleagues, mentors, and leaders in the field tried to warn or discourage him and Virginia from pursuing a career in Alzheimer’s disease research. He recalls their pessimistic outlooks for the field and warnings about the dangers of being lost without direction. They listened, but they also realized that following conventional wisdom or the footsteps of the leaders in the field is also not the answer. They were told that It’s such a swamp, nothing is known about it, but they had a gut feeling that hidden in the swamp was a treasure box that held the secrets and the missing pieces of the puzzle of NDDs. Their work over the years led to the discovery of many secrets and treasures that eventually lured more people to the swamp, which over time led to its transformation it into a flourishing garden.

“We asked our mentors, who were senior like me now 30 years ago, ‘Is this something we should do?’” said Trojanowski. “They all said, ‘No. It’s a swamp, and you’ll ruin your careers because so little is known.’ What they saw as a swamp, we saw as a huge challenge and opportunity that has led to an engaging career^4^.”—John Trojanowski

## Turning Tau from a bystander to a rock star

Walking into a very crowded field, they decided to take the road least traveled, the ‘Tau road’, which led them to their first treasure box and the discovery of Tau as the primary component of the AD tangles. At that time, the building block of the senile plaques (amyloid-β) was already identified by Glenner and Wong, and people’s attention then turned to identify the building blocks of the second pathological hallmark of AD—the neurofibrillary tangles. Although some people had suspected Tau as a potential candidate, John and Virginia were eventually the first to succeed in isolating paired helical filaments from the tangles and demonstrate that they are composed of the Tau protein.

“I was working on a group of brain proteins that form normal neurofilaments that are very abundant in neurons, and so at that time, it was thought that these might possibly be the disease proteins in Alzheimer tangles, but that turned out not to be the case, so, we asked then what forms these tangles?” recalls Lee. “We wanted to collaborate, and identify the proteins that form the abnormal filaments in Alzheimer’s disease tangles, and we decided to go after this—to try to purify the tangles… Using brains we had collected in our center, we were able to demonstrate that Alzheimer tangles indeed were formed by abnormal tau proteins.“^3^—Virginia Lee

“There were many people who had pointed to tau as a candidate, but it was our partnership that led to our success by combining the neuropathology with Virginia’s ingenious development of a way to isolate the paired helical filaments that form neurofibrillary tangles and then sequence these pathological structures to show that the building blocks of these filaments were tau.”^3^—John Trojanowski

They felt that Tau was neglected and did not get the respect it deserved for no good reason. They became vocal challengers of the amyloid hypothesis, which in its early versions proposed that amyloid formation is the primary event responsible for triggering a cascade of events, including Tau tangle formation, that eventually lead to neurodegeneration and AD.

“Tau was quite the controversial discovery, as many researchers at the time felt that the beta-amyloid peptide was the answer to unlocking the secrets of Alzheimer’s. The differences between the two schools of thought were likened to a holy war, between the “BAPtists” (those who espoused the beta-amyloid protein theories of Alzheimer’s) and the opposing “Tauists.“^3^—John Trojanowski

They later discovered that Tau pathology is found in many other brain disorders. To them, this was an exciting discovery that further supported their hypothesis of Tau as a central play in NDDs. However, they continued to face skepticism, and the amyloid hypothesis remained the dominant hypothesis and one that received the most funding.

**“**Tau had a bad reputation of being a bystander and not a real player. It was very difficult to get funding… People said if it is in so many diseases, how can it be important? Maybe it is just a reaction to damage going on in the brain.”He continued, “This was in the early days of neurodegenerative disease research”^5^ –John Trojanowski

The discovery of Tau gene mutations in frontotemporal dementia and Parkinsonism linked to chromosome 17 (FTDP-17) was a game-changer and gave John and Virginia more confidence and tools to defend Tau. But even this was not sufficient to convince the BAPtists. In 1998 he complained that the neuroscience program at the annual society of neuroscience meeting did not have any session on Tau, but expressed great confidence that this trend was coming to an end and the world would not be able to ignore Tau and other proteins. John was always ahead of the crowd.

“…if you look at the neuroscience program this year, you’ll find no sessions on tau. For the last 5 or 6 years we have fallen into “AD: other” and I think next year you’ll see at neuroscience a lot about synuclein, a lot of tau and there’ll be tau session I, II and III because it’s just got to be a focus”^1^ –John Trojanowski

Their discoveries, persistence, and the tools they developed helped pave the way for future discoveries implicating Tau in the pathology and pathogenesis of several other neurodegenerative diseases. This, combined with the continued failure of the amyloid clinical trials and the converging evidence pointing to changes in Tau levels and pathology as better predictors of cognitive decline, led to the transformation of Tau from a bystander to a rock star. It took decades, but it was worth the fight for John and Virginia.

“The religious play on words for these competing hypotheses of what led to brain degeneration in Alzheimer’s disease was a cute thing, but it really did verge on the intensity of religious wars—it could get very harsh. But over the years that has changed. There were people who said that A-beta explains everything, and then when it didn’t, they said, maybe A-beta is necessary but not sufficient; it’s the trigger… So then you have to think of a bullet in a gun. You pull the trigger and the bullet goes out, but if you start trying to fix the trigger, that’s not going to do anything about where that bullet is heading. So if jiggling A-beta doesn’t cure the disease, that’s because it may be the trigger and not the bullet that actually kills neurons in Alzheimer’s disease”^3^ –John Trojanowski

Their journey with Tau inspired them to travel to other uncharted territories and to discover and champion the case of other proteins that later emerged as central players in other neurodegenerative diseases, such as alpha-synuclein in Parkinson’s disease (PD), Multiple System Atrophy (MSA) and synucleinopathies, and TDP-43 in ALS and TDP proteinopathies.

Among their major scientific accomplishments are the following.

- The identification of Tau as the main building block protein of neurofibrillary tangles, one of the two neuropathological hallmarks of Alzheimer’s disease (1991).
- The co-discovery of alpha-synuclein as the primary component of Lewy bodies in PD (in collaboration with Michel Goedert and Maria Grazia Spillantini in 1997) and in glial inclusions in MSA (1998).
- The identification of TDP-43 as the main building block protein of the proteinaceous deposits found in the brain of patients with amyotrophic lateral sclerosis (ALS) and frontotemporal degeneration (FTD) (2006).
- Developing the first-generation models for several neurodegenerative diseases.
- Developing next-generation models to investigate pathology spreading in neurodegenerative diseases by “*tricking the normal proteins into making pathology and spread in the brain*”—Virginia Lee.
- Developing animal models that recapitulate neuropathological diversity and heterogeneity of neurodegenerative diseases.
- The advocacy and efforts to increase awareness about NDDs and more funding for research on NDDs and aging.

## Embracing and championing the complexity of NDDs

John and Virginia were among the first to propose that there is more to AD than just amyloid and Tau. They urged other scientists to explore the role of other proteins like alpha-synuclein and TDP-43 and embrace other hypotheses, including the role of inflammation in NDDs.

“We are not so narrowly focused in our research, and we also work on β-amyloid, very seriously. We also work on α-synuclein. We’d work on more if we had enough brain space to deal with all the molecules but you can’t work on everything and so we have to focus on a couple of things.”^1^—John Trojanowski

Over the years, they continued to explore the complex interplay between different pathologies and how the presence of co-pathologies influences the development and progression of different NDDs. While therapeutic developments in NDDs today remain focused on targeting one protein or pathway at a time, already years ago, John and Virginia predicted that future therapies would require combination therapy approaches that target not only amyloid plaques and tangles but also Lewy bodies and TDP-43 pathologies. A reality that the field and pharma are still reluctant to accept, but one that we cannot escape.

“With Alzheimer’s, you have a whole head full of plaque, tangle, Lewy body, and TDP-43 pathology that needs treatment options, and those options have to address both the plaques and the tangles in all Alzheimer patients, as well as Lewy body and TDP-43 pathology in a large subset of Alzheimer patients.”^4^—Virginia Lee

For the past decades, researchers in the field of NDDs have insisted on connecting amyloid-β and Tau to a single pathway. Initially, Tau was downstream of amyloid plaques. Today, Tau aggregation and pathology formation pathways are depicted as parallel pathways to the amyloid pathway, and dotted lines connect both. In 1998, John suggested that insisting to link these two pathways is a reflection of the limitations of our imagination and emphasized that understanding the normal function of these proteins is an essential step to draw the right lines or change some of the dotted lines to solid lines.

**“**What’s the most mysterious, although there’s lots not known about the familial dementing conditions, is how to think about linking tau and Aβ into a single pathological cascade and that could be limited imagination on the part of scientists or, more likely, a lack of sufficient biological understanding of these proteins. So we need to know a lot more basic biology and that’s not an intellectual sandbox for scientists, it really is the basis from which springs forth new hypotheses about how to look at stuff. You need to have more fundamental understanding of the functions of these proteins and what they normally do and what the dysfunctional consequences of those perturbations are for the cell. That requires basic studies that will then lead to clinical applications, clinical studies, drug discovery efforts and so forth.”^1^—John Trojanowski

To some, especially those in the pharmaceutical industry, the possible role of different pathologies complicates things and presents more challenges for drug discovery. Not for John, as he saw this as an opportunity in disguise.

“I use this economic argument to try to persuade pharmaceutical companies that they will not only have a market for Parkinson’s but also for Alzheimer’s if they target alpha-synuclein in Lewy bodies for drug discovery,” Trojanowski explains, “Here’s a market now for 3 million patients: you can treat 1 million Parkinson’s patients and 2 million of the 4-4.5 million Alzheimer’s patients who harbor alpha-synuclein Lewy bodies in their brains as well as A-beta plaques and tau tangles.“^4^—John Trojanowski

## When asked if he is to name one pathway that is the cause of the disease, what would it be?

**“**Well, I think that’s erroneous because AD is not one disease. …it would be wonderful if we could find what I call the soft underbelly of the beast we call AD to which we could direct a therapy and kill this horrendous animal we call AD. We haven’t identified that yet. It may require several different therapies to block plaques, to block tangles. Certainly for sporadic AD.”—John Trojanowski

## Alzheimer’s disease or Alzheimer’s diseases?

“I think the scientific community appreciates that we should think of Alzheimer “diseases” rather than one disease and the public, I hope, will also appreciate that. There are many forms of this dementia we call AD.”—John Trojanowski

## John, the consensus builder

In addition to his efforts to decipher and highlight the complexity of NDDs, John played important roles in various initiatives to build consensus and conceptual and experimental frameworks to reconcile clinical and neuropathological observations, improve classification schemes, and develop neuropathological diagnostic guidelines for NDDs and their subtypes. He co-led and participated in several initiatives and consortia on (1) developing consensus-based nomenclature and pathological classification scheme for common age-related TDP-43 pathology (2018) such as primary age-related tauopathy (PART) and Limbic-predominant age-related TDP-43 encephalopathy (LATE)^6^, (2) diagnosis and management of dementia with Lewy bodies^7^ and multiple system atrophy^8^, and (3) the gold standard for neuropathological changes in Alzheimer’s disease^8^. These efforts helped shine the light on many common but under-recognized conditions that affect the aging population and the need for more sensitive and specific biomarkers to diagnose and distinguish these conditions from other NDDs. He also played leadership and instrumental roles in the launch and development of the Parkinson Progression Markers Initiative (PPMI).

## John was a great educator and communicator

Making science more accessible to the public and improving public understanding of neurodegenerative diseases were not extra responsibilities but always at the core of his mission and part of his daily work. Despite his remarkable achievements, he insisted that his biggest achievement was to create awareness for aging as an area of study or “*just to make noise about aging*,” as he put it.

“Although my major focus as a biomedical researcher is to try to unravel the causes and mechanisms of neurodegenerative disorders such as Alzheimer’s disease, I also am dedicated to the mission of trying to help educate the public about the seriousness of these disorders and what research needs to be done to develop cures for them.” He continued,“…I think it’s very important that the public knows as much about these issues as possible so they share the decision-making about how we proceed to find ways to treat the many neurodegenerative disease for which we have no effective therapies at this time.”^1^—John Trojanowski

## John loved Analogies

### On the mechanism of protein misfolding and spreading (Fig. 2)

“These disorders – Alzheimer’s, progressive supranuclear palsy, corticobasal degeneration – are diseases of protein misfolding, that can be conceptually hard to understand.

I use a little trick – with my agenda that I print out. This is equivalent to proteins. Genes are very important of course, they encode all the proteins. But proteins are the action figures in our bodies, you have to get your proteins to perform their activities as they should. Proteins are the worker bees of our brain.

Insulin is a good example, it is a signalling molecule that engages a receptor that conveys messages into the cell and then the glucose is taken up. If the receptor were misfolded it would not have been able to bind insulin and signal to take up glucose into the cells, so it’s very important that it has that capability to bind without getting scrunched up. Conveying information is the critical part of what proteins do.

So, when protein misfolding occurs, this results in the loss of function, same as if I crumple the piece of paper - I cannot read my agenda. So it’s bad to misfold, because you lose the functionality of this piece of paper. And if I did this for 10 years, or 20 years, and that’s what we think is the time from the first deposits to become symptomatic. I could fill up this whole building with misfolded paper and it would become a fire hazard.

And so that’s what happened with synuclein, tau, A-beta, TDP-43. And the new concept of how disease spreads is that misfolded protein gets out of the dying cell and encounters its normal counterpart. And when it does so, it corrupts the other normal protein, and now we have two misfolded proteins, and it goes on and on and on until the brain is filled with what we call brain trash.”^5^—John Trojanowski

### Neurodegenerative diseases are caused by fatal attractions between normal and abnormal proteins and cause toxicity

**“**Many neurodegenerative diseases including AD are diseases due to “fatal attractions” between normal or “good” proteins that interact abnormally, precipitate in the brain and cause problems leading to the malfunction of brain cells or their degeneration…” He continued, “…to be more specific, the notion of fatal attractions is something I think links all of these diseases because these proteins somehow become sticky, bunch together and form aggregates, accumulations of proteins, like a cat unstringing a ball of twine and making a mess in a house where it would trip people up and so forth. I think another analogy one could use, is one that I gave them for the article, and that is the notion that by aggregating, the proteins “gum up” the functions of the cell by blocking the normal movement of organelles and proteins around the cell, which is very fundamental and necessary to the function of a cell much in the way that the flow of traffic in LA is necessary for the city to function. Thus, when the traffic is slowed down, this will bring the city to its knees.”^1^—John Trojanowski

### Heredity vs. environment

“People get hung up on heredity vs environment when their border-zones may not be so clear. For example, think about phenylketonuria, a 100% genetic disease. Fatal and terrible clinical course. But change just one environmental exposure (don’t eat phenylalanine), and an affected person will be fine. So it’s also 100% environmental, right? There are probably similar phenomena in neurodegenerative diseases.”—John Trojanowski (personal communication to Peter Nelson, c.2004)

### Why should society invest more in NDD research?

“We are making choices as citizens about spending our resources and we have to ask ourselves the question, is how we use our precious resources now ethical? Is it right not to apply more resources to the fight against such increasingly common killers such Alzheimer’s, Parkinson’s, frontotemporal degeneration, and Lou Gehrig’s diseases? Is that what we should be doing as a society when we are in the throes of an epidemic of Alzheimer’s that I think will make the horrible natural disasters in Japan pale by comparison? Due to dwindling resources and the growing number of patients, the prospect is real that we will have increasing numbers of homeless Alzheimer’s patients walking the streets of America in the next 20 years…” He continued,”… I think that investing in finding a cure for Alzheimer’s should be a national as well as global imperative because the Alzheimer’s epidemic is global.”^4^—John Trojanowski

**Fig. 2:**
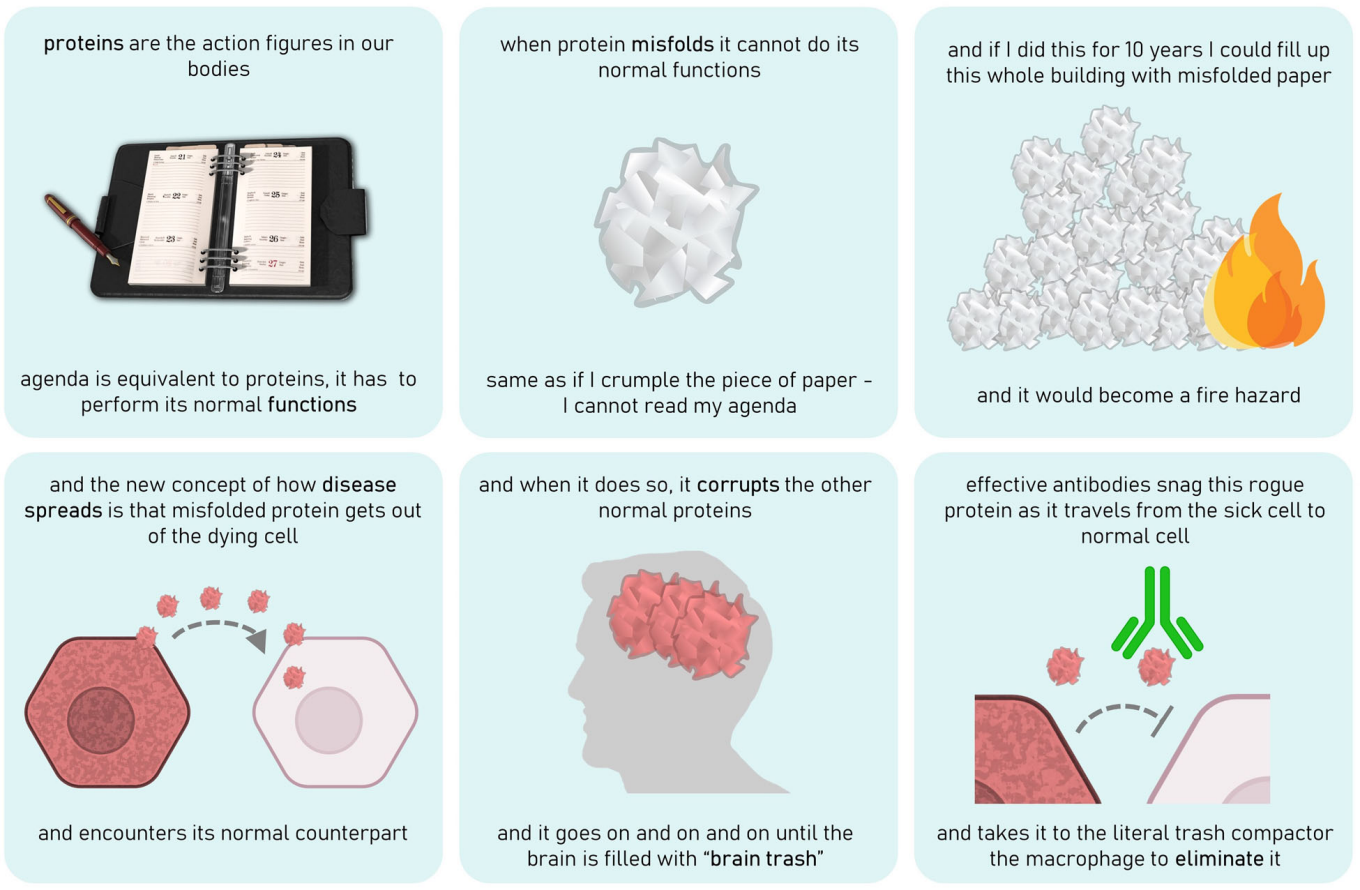
Illustrations of John Trojanowski’s wonderful analogies. Created by Galina Limorenko.

John recognized that tackling Alzheimer’s disease was a monumental challenge. He knew that a cure might not be found in his lifetime, but this did not discourage him from pursuing this goal until his last day in the lab (Fig. 2). His daily missions were to continue adding another knowledge block on the road to the cure, build bridges for collaboration, and strengthen the foundation for translational research and drug discovery in NDDs. He explained why it was important to work on Alzheimer’s disease so eloquently during one of his conversations with his father-in-law, who had expressed skepticism about their plans to pursue a career in AD research.

“The Great Wall of China took 100 years to build. The workers who began at the very beginning of putting the bricks at the bottom of the wall knew they would long be dead before this defense structure was built. Nonetheless, they believed in the mission. They believed in this defense structure. They lived their lives, died, and other workers came along to continue building the wall, so this defense structure was completed.”^9^ –John Trojanowski

I was not among the lucky scientists to be trained by John or to work with him, but I still consider myself one of his students, especially since embarking recently on my journey to rediscover the beautiful and devastating complexity of NDDs. His work and appreciation for complexity continue to inspire me. I have had the pleasure to meet John on several occasions and hosted him and Virginia in Lausanne a couple of years ago. They invited me to visit their laboratory and give a talk in 2017. It was my first time to spend significant time with both of them discussing science and hearing them tell their story and John very proudly speak about the success of their center and, most notably, the success of their students and post-docs. I remember him talking very proudly about their public and patient engagement activities. He gave me brochures about the center and a CD of the PBS documentary he participated in for PBS (Alzheimer’s Disease: Facing the Facts). Their energy, enthusiasm, and passion were both inspiring and infectious. This is why it is difficult for me to accept the word ‘loss’. We did not ‘lose’ John because his work and spirit are alive and continue to inspire us.

## Memories and impact: “There are things that death cannot touch” Jack Throne

**Fig. 3:**
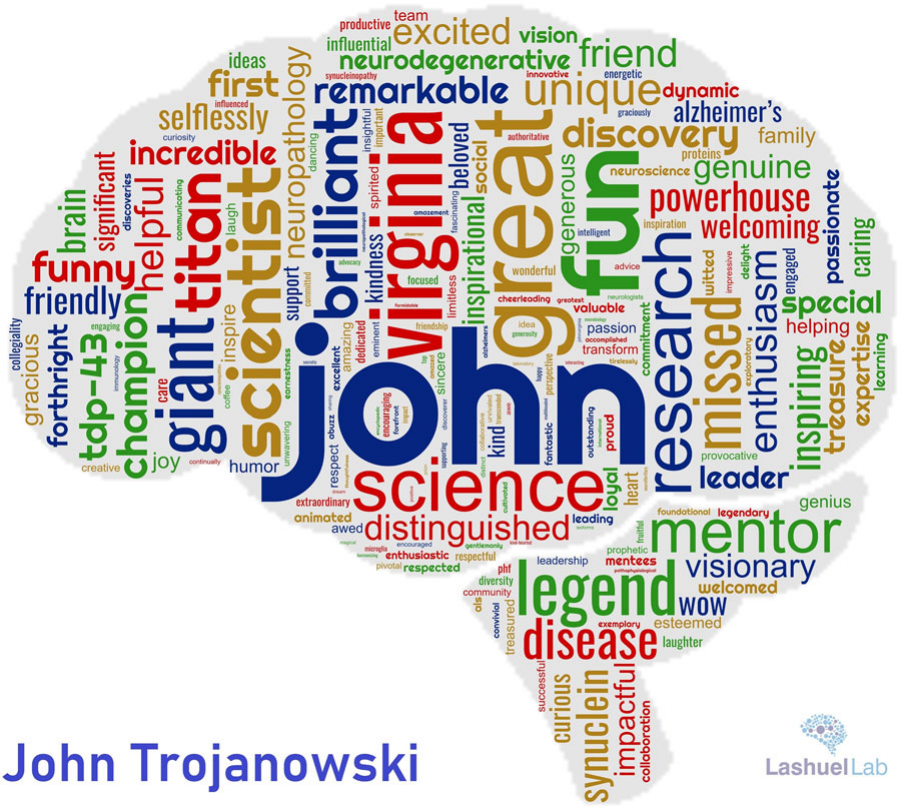
John Q Trojanowski in the words of his colleagues and scientists from the field of NDDs. A John Trojanowski word cloud created using testimonies from his colleagues and members of the neurodegeneration scientific community.

Despite his impressive track record of 100 s of publications, an h-index of 247, more than 260,000 citations, and 100 s of successful trainees, his friends remember him most his unique contributions to the field and their lives, his unique personal qualities, and by how he made them feel about themselves (Fig. 3). Below is a selection of quotes from the statements mourning his death made by leaders in the field of NDDs, colleagues, and friends of John. They were collected from comments published in response to an article by AlzForum mourning the death of John (John Trojanowski, 75, a Giant in the Field of Neuropathology^10^) and comments posted on Twitter.

“Nothing shakes one’s own sense of mortality more than the death of a contemporary. At a time when Tau was still new and shiny, and every day brought a new insight, a time when many of us were meeting each other for the first time at the starting gate of our careers, I visited John and Virginia in their lab at UPenn and their house in Center City.

They were a rarity in science, a team completely knit together. We knew so little then about the disease we studied (and probably know even less now), but their shared lab was abuzz with the promise of all the remarkable discoveries not yet made. The excitement in the lab was palpable as each gel was illuminated on the light box and each brain section came into focus on the multiheaded microscope. The hours raced by filled with animated conversation that I cannot even begin to recreate at this great distance in time. The words are gone but the persona remains, indelible.”—Kenneth Kosik

**“**John Trojanowski was a giant of research on neurodegenerative diseases, and a special friend. He was one of only a handful of scientists at the forefront of research on Alzheimer’s disease (AD) and other tauopathies, Parkinson’s disease (PD) and other synucleinopathies, as well as amyotrophic lateral sclerosis (ALS) and other TDP-43 proteinopathies.

John Trojanowski and Virginia Lee were not only partners in life, but their scientific work was also inextricably linked. We recall a fellow scientist who was surprised to learn that John and Virginia were two different people. He thought John’s name was: Lee Trojanowski. Little did he know! In science it is often important to be able to bounce ideas off others, and some of the discussions between John and Virginia are the stuff of legend; their areas of expertise were beautifully complementary. John and Virginia are an example of the whole being much more than the sum of its parts.”—Michel Goedert and Maria Grazia Spillantini

**“**One trait I suspect some of us recall is John’s ability to ask penetrating questions of a speaker. On occasion, the queries would come one after another, and he might not let up for a while. But this exercise always ended with far more light than fire, and listeners were much enriched by John’s innate facility with the Socratic method

Of course, the defining feature of John’s career was his remarkably successful collaboration with Virginia over more than four decades. The complementarity of their distinct intellects and styles was on display at myriad conferences and academic visits around the globe. Forging such a highly productive and long-lasting partnership is unusual in science, and it is especially impressive between spouses. Lee and Trojanowski, Trojanowski and Lee: These two created a wealth of discoveries and insights that continue to lead us toward a deeper understanding of neurodegeneration.”—Dennis Selkoe

“His style was to always push the field to the next level, whether that was in classic neuropathology and immunostaining or in drug discovery.

His pivotal role, with Virginia, of course, in defining, elaborating, and understanding tau biology led to fundamental discoveries and insights that are central to the field. A true “rock star,” he used his stature to support his colleagues at Penn, contribute to national and international neurodegeneration efforts, and be a wonderful voice of reason in all discussions.

His standing at the microphone at every ADRC directors meeting—jacket slung over his shoulders, ready to challenge an assumption, support great science, or point out a potential problem—will surely be missed. I will personally miss him enormously.”—Bradley Hyman

**“**John was provocative, forthright, and challenging in his comments on the issues. He was a mentor and a major figure, scientifically, in my life in those days, and promoted my career, as he did for so many scientists he worked with. He came committed to helping, deepening, and widening our understanding of the disease process, how to detect it, and how to relate the growing panorama of biofluid and imaging biomarkers to the definitive diagnosis, i.e. autopsy. John’s life partner Virgina continues to reflect their unique “coupleness” as was so very evident at his bedside to the last minute of his life as it was throughout their work together over the years.”—Leslie Shaw

It is absolutely evident that despite the incredible loss, John Trojanowski’s legacy will live on.

“It is hard to forget someone who gave us so much to remember”.

Rest In Peace John

## Supplementary information

Supplemental Material
