# Supplementary material for: Remembering John Q Trojanowski, in his own words: A life dedicated to discovering building blocks and using them to build bridges of knowledge, collaboration, and discovery
Source: NPJ Parkinsons Dis. 2022 Apr 12;8:43. doi: 10.1038/s41531-022-00310-1 (PMC9005626; doi:10.1038/s41531-022-00310-1)
Supplement: Supplementary file 1 — Supplemental Material [file 41531_2022_310_MOESM1_ESM.docx]

**Remembering John Q Trojanowski, in his own words**

**A life dedicated to discovering building blocks and using them to build bridges of knowledge, collaboration, and discovery**

**Hilal A. Lashuel**

Laboratory of Molecular and Chemical Biology of Neurodegeneration, Brain Mind Institute, École polytechnique fédérale de Lausanne (EPFL), Lausanne, Switzerland.

Supplementary Information

**Memories and Impact: “There are things that death cannot touch” Jack Throne**

Below is a selection of quotes from the statements mourning his death made by leaders in the field of NDDs, colleagues, and friends of John. The original comments were published in response to an article by AlzForum mourning the death of John (John Trojanowski, 75, a Giant in the Field of Neuropathology^7^) or posted on Twitter.

**“***He had a piercing intellect and was always a delight to interact with. At many conferences, I remember my conversations with him as being the high point of the whole trip. John’s energy and enthusiasm were boundless and highly infectious. He was always insightful and ready to give his forthright comments and opinions on a wide range of topics. He was also highly collaborative and very generous with his time, providing outstanding mentoring advice.”* –Thomas Wisniewski

**“***In almost any setting, he could be counted on to unreservedly voice his opinion on a wide variety of matters, from NIA funding priorities to ADRC-wide or ADNI-wide initiatives to a discussion of the new research discoveries.*

*He was passionate about science, and his passion drew people to him. He trained and mentored many leading neuropathologists and neuroscientists, in the U.S. and globally, who all remain very loyal to both John and Virginia.”* –John Morris

“*These are shocking news. John was the leader bridging basic with clinical research in neurodegeneration. But more than that he was a very close friend to many of us, always open and interested to discuss science, his own and others. What a loss and sad morning, I miss you already.”* –Mathias Jucker

**“***Over the years, John was a wonderful friend and guide who helped me in so many personal ways. I will be forever indebted to John, and I only hope that I can serve as an equally good mentor and friend to others and in the manner inspired by John.*” –Murray Grossman

**“***An amazing life and long scientific career that of Dr. John Q, Trojanowski. It was a privilege to learn from him at meetings & over drinks. Sending strength & hugs to Dr. Virginia Lee who I am certain will continue the legacy they’ve built together.”* –Malu Tansey

“*In 2012, I was a lost graduate student, recovering from addictions past, holding a bit of a provocative data, the importance of which I lacked appreciation for. At my first conference, I presented the story (poorly), and was praised by a select few (whom I forget). What I won't forget, however, is the thoughtful challenges put forward by John and Virginia. This blossomed into several friendly arguments during the course of the conference. And of course, spirited debates at conferences since. At one point, John tried to mediate a less-friendly argument between a colleague and Virginia. His mediation skills were unrivaled. More importantly, he danced tirelessly. And science was beautiful. It hasn't been the same of late.”* –David Sanders

**“***John was a great teacher and mentored many trainees, many of whom have also made significant contributions to neurodegenerative research. John's legacy is the outstanding science that continually streams from the scientists he trained and from the laboratories and centers that he created and supervised up until the end.”* –Creighton (Tony) Phelps

“*John had amazing energy and passion for science. He seemed to be working all the time, and I often urged him to get some sleep. He was particularly passionate about the multifactorial causes of dementia, and the importance of α-synuclein, TDP-43, cerebrovascular disease, and other pathologies as causes of, and contributors to, cognitive decline and dementia.*

*Aside from his amazing scientific contributions, John inspired generations of medical investigators including pathologists, neurologists, psychologists, and biochemists. For example, Dan Skovronsky, now at Lilly, trained as a neuropathologist with John and Virginia, and then went on to found AVID, which developed Florbetapir, the amyloid PET tracer. Many hundreds of investigators have been inspired, influenced, or directly trained by John.*

*The ADNI team will remember John for his passion for science, and for reminding us that cognitive decline and dementia are caused by more than plaques and tangles, and in this he was obviously prophetic.”* –Michael Weiner

“*John was encyclopedic regarding the neuropathological features of neurodegenerative disorders. He not only codified modern neuropathology, he performed the research that changed the field. I looked forward to every paper that would come from John and Virginia!*

*John had an incredible zeal for science, culture, and life. He and Virginia traveled the world as ambassadors for science and for their beloved University of Pennsylvania. He was a relentlessly curious man who lived every moment of his life with passion.”* –Bruce Miller

**“***John gave freely of his time and expertise not only to his many trainees but to anyone who shared his vision of a better future for people suffering with neurodegenerative diseases. Through his remarkable generosity, many of us have stood on his shoulders, and his impact is in fact much greater than the seminal discoveries and innovations made by the Lee/Trojanowski laboratory.*” –Thomas Montine

*“John’s contributions to our understanding of brain disease undoubtedly have sped the pace of therapeutic development toward novel approaches with potential to stop or even prevent diseases such as Parkinson’s. His work has impacted millions, and his legion of trainees will continue to build on those breakthroughs. We are so grateful for his vision and for his partnership over the years,”-* Todd Sherer

“*A true giant in science. And a warm person who was a great entertainer at every conference dinner. Will be dearly missed.”* –Patrik Brundin

“John Trojanowski was a close friend, a brilliant mind, an enthusiastic, forward-looking scientist. We will miss him deeply!” –Günter Höglinger

Comments by former trainees

“*Working with John and Virginia was a real turning point in my professional life, as it has been for so many other scientists. John was a caring mentor, a fantastic teacher, a great leader, always energetic, enthusiastic, and most importantly, not only a brilliant scientist, but also a fascinating, remarkable, and generous human being.”* –Manuela Neumann

“*I’m just one of hundreds, perhaps thousands, whom John and Virginia influenced by example and by mentoring. They seriously helped launch my career as inspirational collaborators.*” –Barry Greenberg

“*John’s achievements, his partnership with Virginia, his presence at the conference mic stand, and his leadership are legendary. I am left flooded with memories, from his collarless dress shirts and fanny pack to his penchant for ice in his Guinness.*

*I am privileged to have had him as mentor for the last 25 years. His mentorship is evidenced by the many neuropathologists who populate ADRC neuropathology cores or are leaders in our field who continue his life’s work, including Daniel Skovronsky, Andrew Lieberman, Anthony Yachnis, Mark Forman, Peter Nelson, Subhojit Roy, Sriram Venetti, Pallavi Gopal, Aivi Nguyen, Stefan Prokop, and myself.”* –Edward B. Lee

“*John was a lovely person and great scientist. He played a huge role for me as a Penn postdoc, setting me on the right path... as I am sure he did for so many. I will miss him greatly. Thank you, John... RIP.”* –Henry Paulson

“*Got my start as an undergrad at the CNDR & John would sit at the microscope with me to review slides. I got excited about something new I saw and we argued about whether it would be a “Trojanowski Body” or a “Gur Body”. It turned out to be artifact, but he was so gracious about it.”* –Tamar Gur

“*You are my greatest mentor forever...”* –Maiko Uemura

“*I and many others would not be where we are without John's actions and guidance.”* –Li-San Wang

“*We stand on the shoulders of giants, and the world has lost a great one. I met him during an interview and his enthusiasm, curiosity, and kindness would light a fire under anyone. I couldn’t wait to get back to the lab after our conversation. Rest easy.”* –Megan Duffy

“*RIP, John. Your work has inspired many young scientists & impacted many many more patients*” –Jun J. Mao
